# On-farm storage loss estimates of maize in Kenya using community survey methods

**DOI:** 10.1016/j.jspr.2023.102107

**Published:** 2023-05

**Authors:** Hugo De Groote, Francisca Ndinda Muteti, Anani Y. Bruce

**Affiliations:** International Maize and Wheat Improvement Centre (CIMMYT), Kenya

**Keywords:** Maize weevil, Larger grain borer, Storage losses, Maize

## Abstract

Maize is the most important staple in sub-Saharan Africa (SSA), with highly seasonal production. High storage losses affect food security, but good estimations are lacking. A new method using focus group discussions (FGDs) was tested with 121 communities (1439 farmers, 52% women) in Kenya's six maize-growing zones, to estimate the maize losses to storage pests and analyze farmer practices. As control strategies, half of the farmers used chemical pesticides (49%), while hermetic bags (16%) and botanicals (15%) were also popular. Relative loss from weevils in the long rains was estimated at 23%, in the short rains 18%, and annually 21%. Fewer farmers were affected by the larger grain borer (LGB) than by maize weevils: 42% in the long rainy season and 32% in the short rainy season; losses from LGB were also smaller: 19% in the long season, 17% in the short season, and 18% over the year. Total storage loss, from both species combined, was estimated at 36%, or 671,000 tonnes per year. The greatest losses occur in the humid areas, especially the moist mid-altitudes (56%), and with smaller loss in the drylands (20–23%). Extrapolating the point data and overlaying with the maize production map shows the geographic distribution of the losses, with the most important area found around Lake Victoria. FGDs provide convenient and cheap tools to estimate storage losses in representative communities, but a total loss estimate of 36% is higher than is found in other studies, so its accuracy and framing effects need to be assessed. We conclude that storage pests remain a major problem, especially in western Kenya, and that the use of environmentally friendly technologies such as hermetic storage and botanicals needs more attention, both by the public extension service and private agrodealers.

## Highlights

• Discussions were conducted with 121 communities (1439 farmers, 52% female) in Kenya.
• Farmers estimated maize storage loss by weevils (21%) and larger grain borers (18%).
• Maize storage loss was estimated at a total of 36%, especially high in Western Kenya.
• Farmers used chemical pesticides (49%), hermetic bags (16%) and botanicals (15%).
• Environmentally friendly technologies need more attention by policy and extension.

## Graphical abstract

**Figure:**
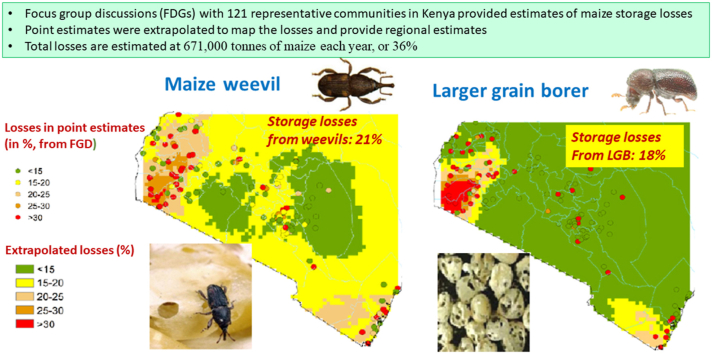

## 1 Introduction

Postharvest losses (PHL) remain a key focus area of discussion, especially in sub-Saharan Africa (SSA), where most households rely heavily on farm produce for their income (Sheahan and Barrett, 2017). Food losses imply reduced availability of food to feed the growing population, particularly in developing countries (Kumar and Kalita, 2017). In SSA storage losses threaten households' food security and undermine their market returns (Midega et al., 2016; Stathers et al., 2013). Farmers commonly indicate storage pest problems as major constraints to their livelihood (De Groote et al., 2004; Likhayo et al., 2016). Reducing postharvest losses has been identified as a sustainable strategy to reduce hunger and improve grain farmers' livelihoods without increasing pressure on the natural environment (Tefera, 2012). However, the estimation of PHL remains controversial. Previous studies have shown that PHL could range from 20% to 40% in African countries (Kumar and Kalita, 2017). However, specific estimates of storage losses in maize due to insect infestations in Africa range from 40 to 50% — loss estimates frequently quoted by the development community and cited in Zorya et al. (2011)— although a recent meta-analysis estimates the range at 4–21%, with the upper limit representing losses without interventions (Affognon et al., 2015). Despite the controversy, scientific studies and national estimates are lacking in many countries, including Kenya. However, good estimates of these losses are needed to target and prioritize interventions, either technologies or policies (Kaminski and Christiaensen, 2014) and to compare their costs and benefits (Affognon et al., 2015).

Some of the common pests associated with this damage are maize weevils (*Sitophilus zeamais*) and the larger grain borers (*Prostephanus truncatus*) (Tefera, 2012). The maize weevil is a pest of economic importance that infests the fields, but causes most damage during storage (Giga and Mazarura, 2011). In Africa the larger grain borer (LGB) was first observed in Tanzania in the late 1970s and from there spread to eastern and southern Africa. The LGB caused severe losses in stored maize, three to four times higher than the losses before its arrival (Farrell and Schulten, 2002). In Kenya the LGB was first reported in 1983 in the Taveta division, which borders Tanzania (Kega and Warui, 1983). Several studies have been conducted on the LGB and the losses that it causes (Boxall, 2002).

Several innovations have been developed to reduce losses from storage pests, including chemical pesticides specifically designed to control storage insects, such as actellic (Urono, 1999) and super actellic (Stathers et al., 2008). Alternatives that pose less risks to the environment and human health include botanicals (Eticha and Tadesse, 1998; Isman, 2006) and hermetic storage such as metal silos (Tefera et al., 2011) and hermetic bags (Quezada et al., 2006). Hermetic technologies were found to be effective on station (De Groote et al., 2013) as well as on-farm (Ndegwa et al., 2016). Hermetic bags were potentially profitable if farmers stored maize for four months per season and the bags lasted four years (Ndegwa et al., 2016).

Despite the broad literature on storage pests that often describes their spread and provides estimates of the losses that they cause, scientifically sound and nationally representative studies to estimate losses and assess economic impact are rare, and so far, none have been conducted in Kenya. Close monitoring could help determine the losses caused by storage pests, especially the common maize weevil and LGB that cause substantial losses. However, scientific and systematic observations of storage losses on a national scale would be expensive. An alternative approach is the use of farmer surveys, for example the Living Standards Measurement (LSM) surveys. In some countries these have included estimates of storage losses, for example in Ethiopia (Hengsdijk and de Boer, 2017), and Malawi, Uganda, and Tanzania (Kaminski and Christiaensen, 2014). However, individual farmer surveys are expensive, while estimates obtained from systematic and representative Focus Group Discussions (FDGs), on the other hand, are much more economical and also provide good results. This has been shown with studies on maize lethal necrosis (MLN) (De Groote et al., 2016, 2021) and the fall armyworm (FAW) (De Groote et al., 2021).

In this study we used focus group discussions conducted in randomly selected communities that were representative of the different maize agroecological zones (AEZs) in Kenya to assess storage losses. Specific objectives of the study were: i) to assess farmers' knowledge of maize weevils and LGBs and their observations of both pests in the two seasons before the survey; ii) to estimate the proportion of farmers affected by both storage pests and the percentage of maize lost in storage on affected farms; iii) to estimate the total relative loss on all farms (as a percentage) by multiplying these two variables; and iv) to extrapolate the results to estimate the total loss (in tonnes) in each of the six agroecological zones of Kenya and for the whole country.

## 2 Materials and methods

### 2.1 Storage loss estimation

Storage loss is defined as the difference in maize quantity at the beginning of the storage period *Y*_*0*_ and at the end, *Y*_*t*_*,* expressed as a proportion or percentage

(1) $$r=\frac{\left(Y_{0}-Y_{t}\right)}{Y_{0}}\left(\times 100\right)$$

Instead of trying to measure *Y*_*0*_ and *Y*_*t*_ directly, we asked farmers during group discussions to estimate the proportion of farmers affected by storage insect pests (*F*_*a*_) in their community and the estimated storage loss (in %) experienced by the affected farmers (*r*_*a*_). Relative loss among all farmers in the community was then calculated as

(2) *r = F*_*a*_*x r*_*a*_

As the communities were selected randomly from the major maize production zones, average storage losses can be multiplied by the estimated maize stored in each zone to estimate maize quantities lost. The quantity of maize stored is the quantity produced minus the amount marketed. Using group discussions for pest problems was first used in Kenya in 2000 to assess the importance of different maize pests (De Groote et al., 2004), and later to quantify crop loss and distribution from maize lethal necrosis (MLN) disease in 2013 (De Groote et al., 2016) and FAW in 2018 (De Groote et al., 2020). In this paper we expand the methodology of using FGDs to quantify pest losses due to storage pests.

### 2.2 Study design and site selection

**Fig. 1:**
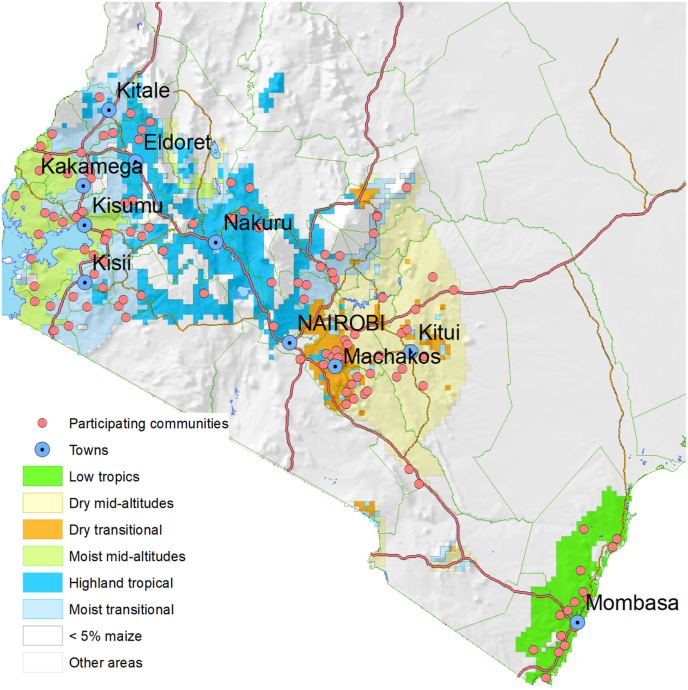
Map showing the agroecological zones and sites of the FGDs.

A community survey was executed in Kenya in 2018, with a design very similar to the community survey of 2013, from which the losses due to maize lethal necrosis (MLN) were estimated (De Groote et al., 2016). The 2018 study targeted the same 121 communities interviewed in 2013, who had been randomly selected to represent Kenya's six main maize production areas (Fig. 1). The primary purpose of the 2018 community survey was to assess farmer prioritization of various stresses and measure the impact of these for the Stress Tolerant Maize for Africa (STMA) project, and was driven by the sudden arrival of the fall armyworm (FAW). Data were collected through focus group discussions (FGDs), and the results for other pests have been presented elsewhere, in particular for MLN (De Groote et al., 2021) and FAW (De Groote et al., 2020).

### 2.3 Development of survey tools

CIMMYT contracted Agri-Food Economics Africa, a research company based in Kenya, to undertake the study. The development of the questionnaire was a consultative process undertaken during the first half of 2018, involving CIMMYT and its partners who had a special interest in the FAW: the International Centre of Insect Physiology and Ecology (ICIPE), the Food and Agriculture Organization (FAO), and the CAB International (CABI), as well as CIMMYT economists and entomologists. Comments from these partners were considered, and efforts were made to harmonize sections of the tools with those of the partners, such as FAO's FAW modules.

The study's primary goal was to assess the importance of different maize production and storage stresses, as perceived by farmers in the different agroecological zones where maize is produced. A draft questionnaire was developed and tested, with separate modules addressing different pests, including MLN, FAW, maize stem borer, and two storage pests: the maize weevil and the larger grain borer.

The questionnaire was pre-tested for two days in June 2018, with two communities in Embu County not participating in the survey. In addition to economists from CIMMYT and Agri-Food Economics Africa, a CIMMYT entomologist and an economist from ICIPE participated in the pre-test. After the pre-testing, adjustments were made, and the questionnaire was uploaded onto the Survey CTO platform. After two days of enumerator training, the survey was piloted in Murang'a County, followed by a recap to raise and discuss all the issues observed. The researchers discussed all the additional issues observed during training and piloting and finalized the questionnaire for data collection.

**Fig. 2:**
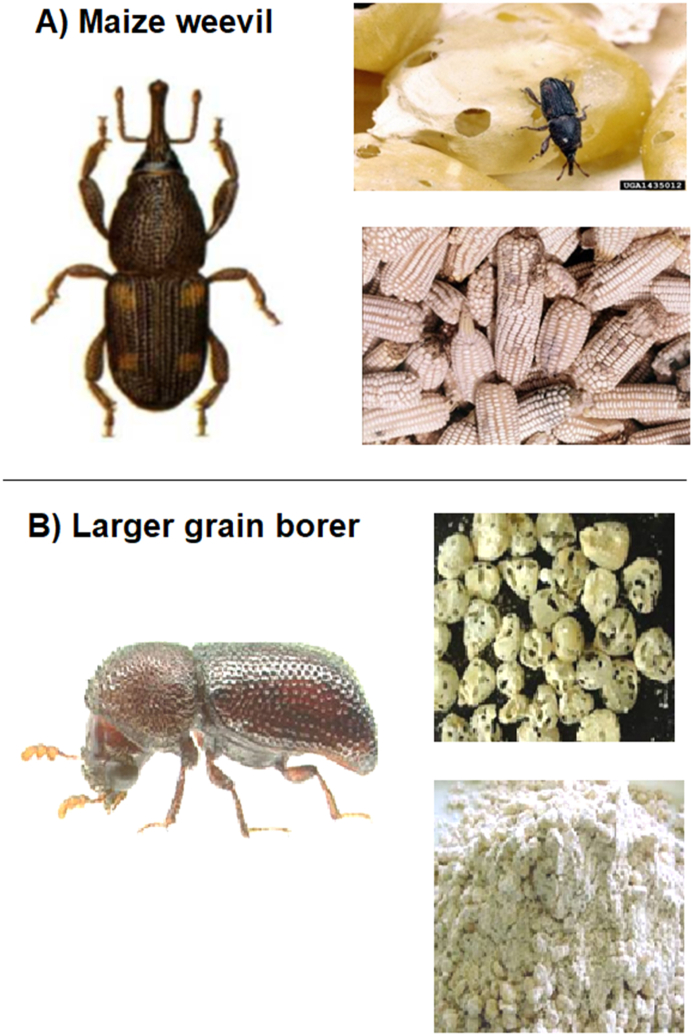
Pictures shown to participants to determine if they could correctly identify maize weevil (Panel A) (Mofokeng, ARC) and larger grain borer (Panel B) (provided by Georg Georgen, International Institute of Tropical Agriculture).

To discuss the different pests with the participants in the group discussions, photographs of the pests were used (see pictures for the storage pests in Fig. 2) and shown to farmers. The photos also provided a way to gauge farmers' awareness and knowledge of the weevils and larger grain borers. CIMMYT entomologists and pathologists assisted in gathering these pictures and in refining the descriptions of the various stresses. The final version of the images was printed and laminated for use in data collection.

### 2.4 Data collection

Data collection was undertaken by Agri-Food Economics Africa, which recruited two teams, each consisting of an experienced supervisor and two experienced enumerators. All team members were adequately trained in all aspects of the survey and questionnaire and participated in the survey pilot as part of the training and preparation. Data collection took place over 41 days, from June 18th to July 28th. Each field team was given a list of the communities to interview, with the previously allocated identification number, location details (division, location, and sub-location), and contacts of the members who participated in the 2013 FGDs. The contacts in the communities, usually a leader from a farmer group or the local administration, were each asked to invite between 10 and 15 maize farmers. A more detailed description of the exercise is provided in the previous paper on FAW (De Groote et al., 2020). At the end of the data collection, all the targeted 121 communities were interviewed, representing 100% coverage with no replacements, and 1439 farmers participated, of which 742 were women. In each community, farmers were asked to estimate the proportion of farmers affected (*F*_*a*_*)* and the relative losses among affected farmers (*r*_*a*_*)*, both for weevils *(w)* and for LGB *(g)* and for both the long (*1)* and the short rains (*s).* All survey data were uploaded to the CIMMYT repository (De Groote et al., 2022).

### 2.5 Analysis

Multiplying the proportion of farmers affected with the relative loss among affected farmers led to estimates of relative loss (*r*_*ijk*_*)* by insect species (*i), by* season (*j)* and by AEZ (*k)*. To estimate relative losses over the whole year, a weighted average was calculated using the maize production in each season as weights, for each species (*i)* and each AEZ (*k)*:

(3) $$r_{ik}=\sum_{j}w_{jk}r_{ijk}$$

where *w*_*ik*_ is the proportion of maize produced in season *j* for AEZ *k.* Seasonal weights for each season were obtained from the 2013 CIMMYT household survey (Wainaina et al., 2016). Zonal weights were obtained from calculating the zonal maize production by overlaying the map of maize production zones (Hassan et al., 1998) with the 2017 SPAM maize production map for Africa (IFPRI, 2020) (Table 1).

**Table 1:** Maize agroecological zones in Kenya, with estimated maize area and production in 2017.

Agroecological zone	Sample size (communities)	Elevation	Maize 2017^a^			Population^b^	Weights			
	N	(masl)	Area (1000 ha)	Production (1000 tonnes)	Yield (t/ha)	(1000)	Area	Production	Population	Maize production long rains^c^
Lowland Tropics	15	0–700	58	37	0.65	2857	0.03	0.01	0.06	0.621
Dry Mid-altitude	17	700–1400	401	196	0.49	3825	0.19	0.06	0.08	0.415
Dry-Transitional	18	1100–1700	588	486	0.83	5403	0.28	0.15	0.12	0.510
Moist-transitional	32	1200–2000	386	524	1.36	7931	0.19	0.16	0.17	0.738
Highlands	20	1600–2900	248	586	2.36	1801	0.12	0.18	0.04	0.990
Moist Mid-altitude	19	1110–1500	103	109	1.06	12,137	0.05	0.03	0.26	0.608
Total maize zones	121		2086	3186	1.53	45,890	1.00	1.00	1.00	0.86

Sources: ^a^ SPAM 2017, ^b^ WorldPop., ^c^ CIMMYT's 2013 household survey (Wainaina et al., 2016).

Total relative loss per species *i* was calculated again by weighted average over the AEZs:

(4) $$r_{i}=\sum_{k}w_{k}r_{ik}$$

where *w*_*k*_ is the proportion of annual maize production in AEZ *k.*

Absolute losses, or the quantity of actual maize lost to each species *(i)*, were calculated by multiplying the relative loss $r_{ijk}$ with the respective production *P*_*jk*_ of season *j* and AEZ *k*:

(5) $$L_{ijk}=r_{ijk}\;P_{ijk}$$

To calculate total storage loss over both species we needed to avoid double counting, as one insect cannot cause losses in the part that is already lost through the other species. If the amount of maize stored is *Y,* and the loss caused by weevils is calculated first as *r*_*w*_*Y,* then the amount lost to LGB can be calculated as *r*_*l*_ *Y (*1-*r*_*w*_*).* The total amount lost (absolute loss) is then:

(6) *L*_*t*_ *= Y(r*_*w*_ *+ r*_*l*_*- r*_*w*_*r*_*l*_)

The total relative losses are thus:

(7) $$r=r_{w}+r_{w}-r_{w}r_{g}$$

For geographic analysis, we extrapolated the annual relative losses for weevil, LGB and combined using kriging with the software ArcMap (REF) to create maps of relative losses. We overlayed the combined map of relative losses with the SPAM 2017 grid map of maize production (IFPRI, 2020) and multiplied the two layers to obtain a map of absolute losses.

## 3 Results

### 3.1 Knowledge and recognition of maize weevil and LGB by communities

**Fig. 3:**
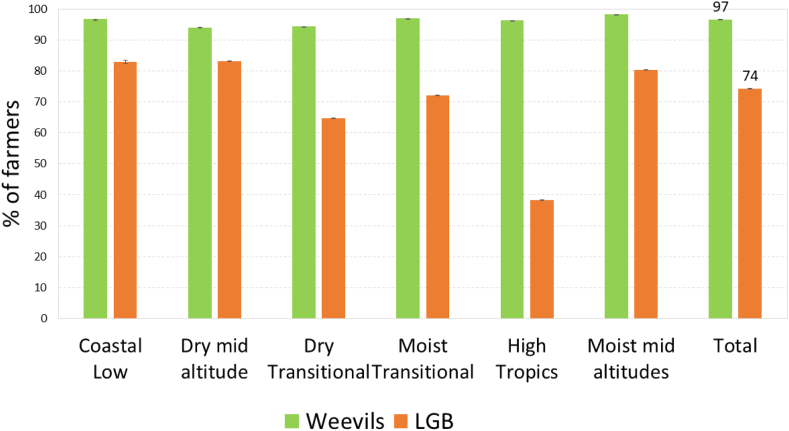
Percentage of farmers who correctly identified maize weevils and LGB.

At the beginning of the focus group discussions, participants were shown pictures of the maize weevil and the LGB (Fig. 2) and asked if they could recognize the pests. Almost all participants (97%) could recognize the maize weevil, and most participants (74%) could also recognize the LGB (Fig. 2). There was hardly any variation between regions for weevils, but substantial variation for the LGB. Weevils were most recognized in the moist mid-altitudes (98%) and least in the dry mid-altitudes and dry transitional zones (94%). For LGBs, recognition was highest among participants in the coastal lowland and dry mid-altitudes (83%) and lowest in the high tropics (38%), which is linked to the relative importance of the pest in these zones (Fig. 3).

### 3.2 Occurrence and spread of the maize weevil and LGB in maize-growing regions

**Fig. 4:**
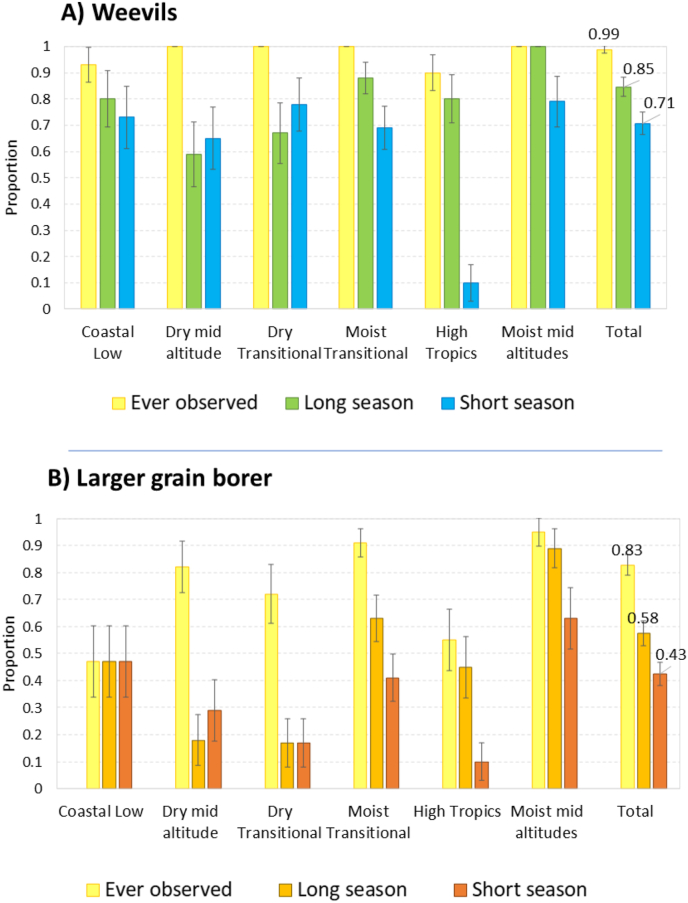
Proportion of communities that observed maize weevils and LGB in the different maize growing regions, at least once or over the last two seasons of 2017.

After identifying the two storage pests, weevils and LGB, participants were asked whether they had ever observed them in their community, and if they had observed them during the last two seasons, the long and short rainy seasons of 2017. All communities but one (99%) reported that they had observed maize weevils at some point in time (Fig. 4). Observation of maize weevils was widespread in all zones: in each AEZ, weevils had been observed by at least 93% of communities. Most communities (85%) had also observed weevils during the last long rainy season, but fewer (71%) during the last short rains. Weevils in the long rains were observed in all zones and observed by at least 59% of the communities (this was the lowest level, in the dry mid-altitudes). During the last short rainy season, weevils were also observed by most communities in all zones except for the high tropics, where it was only observed by 10% of communities. This is understandable as there is hardly any maize grown in the highlands in the short season.

The LGB had also been observed by most communities (83%), although not as frequently as weevils. A majority of communities had observed them during the long rains of 2017 (58%), but less than half during the last short rains (43%). During the long rains, observations of LGB varied from almost all communities in the moist mid-altitudes (89%) to about half at the coast (47%). The LGB was clearly less important in the short rains; it was observed by more than half of communities in the moist mid-altitudes (63%), but by less than half the communities in the other zones, down to 10% in the highlands.

The results also show that storage pests are more important in the long rains, except for the drylands, where pests (and maize production) are more important in the short season.

### 3.3 Farmers affected and relative losses caused by storage pests

**Fig. 5:**
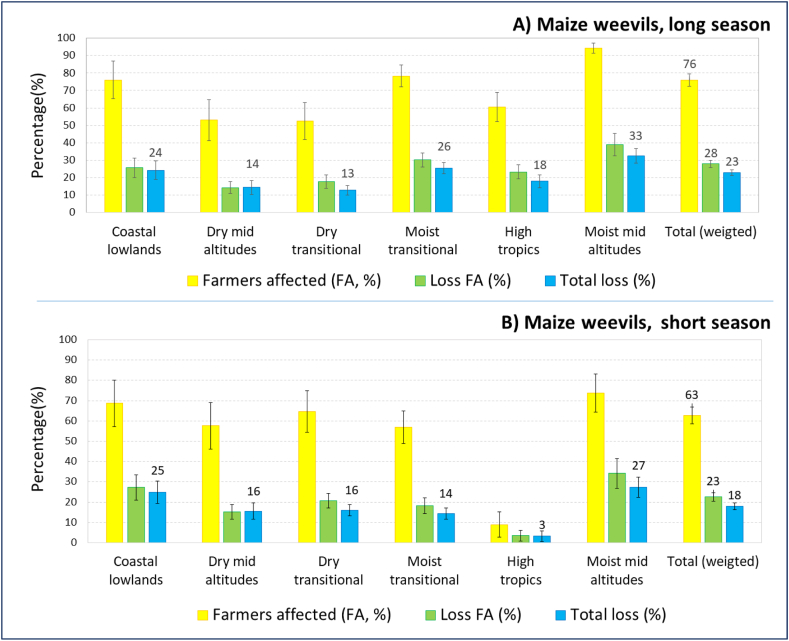
Farmers affected (%), loss on affected farms (%) and total loss from maize weevils.

Next, participants were asked to estimate the percentage of farmers in their communities affected by the pests, and the percentage loss among affected farmers. Maize weevils were more important during the long rainy season, when three quarters of farmers were affected (76%), than in the short season, when two-thirds (63%) of farmers were affected (Fig. 5). Loss among affected farmers was also greater in the long rains (at 28%) than in the short rains (23%). Similarly, loss from maize weevils among all farmers, calculated by multiplying the percentage of farmers affected with the loss among affected farmers, was also higher in the long rains (23%) than in the short rains (18%). There were substantial differences between zones, and hot and humid zones were more affected by maize weevils than dry and colder zones. Losses among all farmers were particularly low in the drylands (13% for dry transitional and 14% for dry mid-altitudes) and high in the humid areas (26% in the moist transitional and 33% in the moist mid-altitudes). In the short season, losses from maize weevils among all farmers were still highest in the moist mid-altitudes (27%), but also at the coast (25%). Losses were higher in the short season in the drylands (16%), where the short season is more important, but there were almost no losses in the highlands, where only a little maize is produced and stored during that season.

**Fig. 6:**
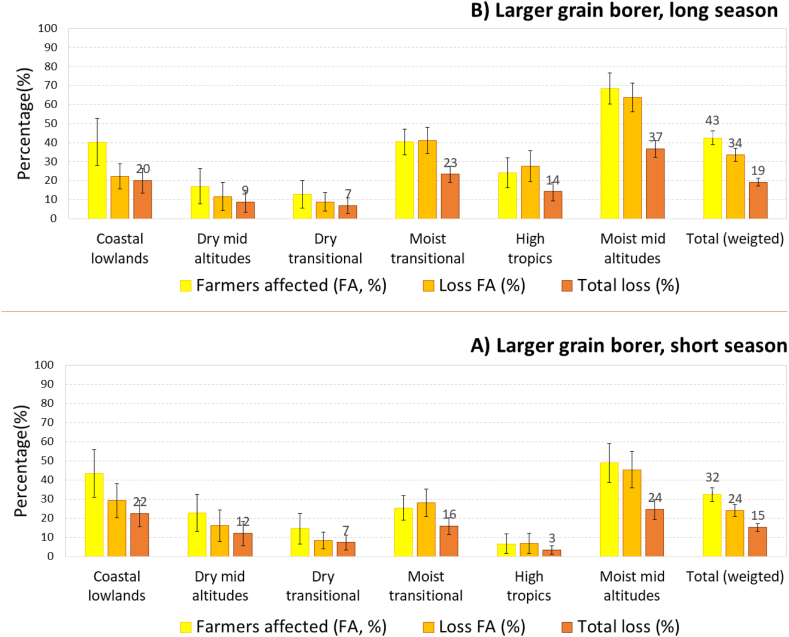
Farmers affected (%), loss on affected farms (%), and total loss from larger grain borer (LGB).

For the larger grain borer, less than half of the farmers were affected (42% in the long season and 32% in the short season), but the losses among affected farmers were larger (37% and 29%), so losses among all farmers were similar to those from weevils: 21% in the long season and 17% in the short season (Fig. 6). Differences in losses caused by LGB between zones were similar to those for weevils but more pronounced; in the long rains, losses from LGB for all farmers ranged from 7% in the dry transitional zone to 37% in the moist transitional zone, while in the short season they ranged from 3% in the highlands to 24% in the moist mid-altitudes.

### 3.4 Total annual relative losses caused by the maize weevil and LGB

**Fig. 7:**
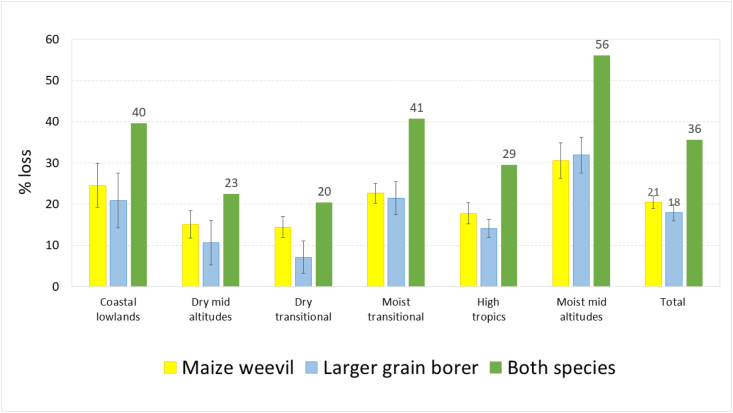
Annual relative loss from weevils and LGB (%).

Using weighted means, annual relative losses were estimated at 20.5% for the maize weevil and 17.9% for the LGB (Fig. 7). Losses to both pests were highest in the warmer and humid areas, in particular the moist mid- and transitional altitudes and the coastal lowlands, and lowest in the drylands and high tropics. Total relative losses from both species, based on farmer estimates, were calculated using Equation (7); the results indicate that more than a third of maize stored (35.7%) is lost to storage pests, ranging from a fifth in the dry mid-altitudes to more than half in the moist mid-altitudes.

As the storage losses are higher in moist zones and lower in dry zones, we explored the link between losses and climate through correlation analysis (Table 2). We found a strong correlation between losses from the two species with both relative humidity and temperature during the short season (>0.3), but not during the long season. Moreover, the two climate variables were highly correlated (0.8) and incorporating them into a regression model did not yield significant coefficients.

**Table 2:** Correlation between relative storage losses (in %), temperature (°C) and relative humidity (%).

Species	Season	Relative humidity (%)	Temperature (°C)	Losses from the other species (LGB and weevils respectively)
Losses from weevils (%)	Short	.314**	.316**	.518**
		.000	.000	.000
	Long	−.002	.088	.400**
		.980	.337	.000
losses from LGB (%)	Short	.214*	.223*	.518**
		.912	.119	.000
	Long	−.026	.174	.400**
		.779	.056	.000
Relative humidity (%)	Short		.842**	
			0.000	
	Long		.820**	
			0.000	

Significance levels: * = P ≤ 0.05, ** = P ≤ 0.01, *** = P ≤ 0.001.

**Fig. 8:**
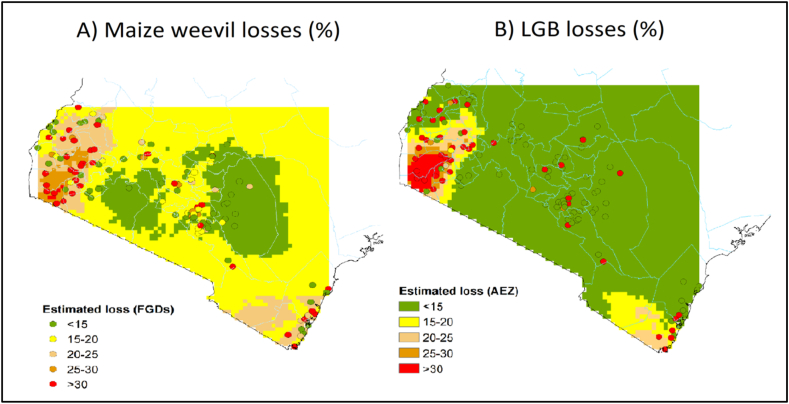
Geographical distribution of relative losses from maize weevils (left) and larger grain borer (LGB) (right).

Finally, extrapolation of the point estimates of relative losses provided a map with the geographic distribution of relative losses for each storage pest (Fig. 8). The circles represent the georeferenced communities, and the different colors show the level of relative losses, extrapolated from the point data. The map shows two distinct zones of high relative storage losses, both with high humidity and temperature: the first in the west, around Lake Victoria, and the second, with somewhat lower losses, in the south from the coast inwards to Taita Taveta. The areas with high losses from weevils and LGBs largely overlap, with the LGB showing more contrast, with small areas with high losses of over 30%, and large areas of low losses. The area in between the high loss zones is relatively less affected, although with substantial variation; the red dots indicate communities with high losses, even in green areas with generally low losses.

### 3.5 Absolute losses

To estimate absolute losses — the actual quantities of maize lost — we multiplied the total annual relative losses for each AEZ (Fig. 7), with maize production calculated for each AEZ from SPAM 2017 (Table 1), using Equation (6). The calculations showed that each storage pest caused similar absolute losses: about one-fifth of production or almost 400,000 tonnes each year, with losses from maize weevils slightly higher (Table 3). In all maize production zones combined, a total of 671,000 tonnes per year were lost in storage due to insect pests. Almost half the loss occurred in the moist mid-altitudes (45%) and most of the rest (43%) in the high potential zones, equally divided over the moist transitional and the high tropics. The marginal zones, especially the lowlands and the dry transitional zone, accounted for only a small proportion of overall absolute storage losses in the country.

**Table 3:** Absolute losses from weevils an LGB in storage (quantities in 1000 tonnes, by season and annual).

Zone	Absolute storage losses (1000 tonnes)						
	Weevils			LGB			Combined
	LR	SR	Annual	LR	SR	Annual	Annual
Coastal lowlands	5.6	3.5	9.2	4.6	3.2	7.8	15.1
Dry mid-altitudes	11.7	17.9	29.6	7.1	2.1	9.3	37.4
Dry transitional	31.8	38.4	70.2	17.3	7.4	24.8	91.4
Moist transitional	99.0	19.7	118.6	90.5	89.7	180.2	258.0
High tropics	103.9	0.2	104.1	82.6	1.3	84.0	173.1
Moist mid-altitudes	21.7	11.8	33.4	24.4	66.0	90.4	96.1
Total	273.7	91.4	365.1	226.6	169.7	396.3	671.2

**Fig. 9:**
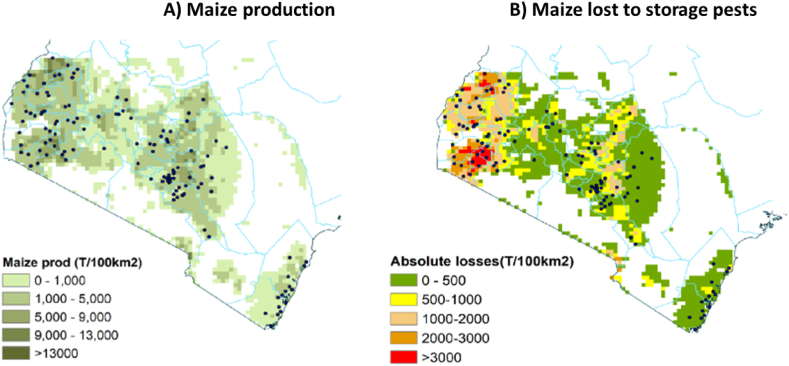
Geographical distribution of maize production (in tonnes/100 km^2^, Panel A) and quantity of maize lost caused by storage pests, from maize weevils and LGB combined (in tonnes/100 km^2^, Panel B).

Finally, we multiplied the grid map of relative losses (Fig. 8, combining panels A and B using Equation (7)) with the grid of maize production (Fig. 9, Panel A) using Equation (6), which resulted in a grid map with the quantities of maize lost (Fig. 9, Panel B). The map shows two distinct areas of high absolute losses: the most important one is in western Kenya, around Lake Victoria, with large areas that suffered maize losses above 35 tonnes per km^2^. A second area of large absolute losses is found in the dry areas of eastern Kenya, roughly from Machakos to Meru, with losses of 10–20 tonnes/km^2^ (Fig. 9).

### 3.6 Farmers’ control strategies

Participants of the study reported eighteen different control methods that they used against storage pests, grouped by us into five major categories: chemical, mechanical, traditional, botanical, and natural methods (Table 4). The most popular method was chemical insecticides, used by almost half of the communities (49%), followed by hermetic bags (16%). Botanical methods were used by 15% of communities and included treatments with pepper, neem, and ferns (each used by less than 10% of communities). Traditional methods were used by 10% of communities and included heat treatment, and smoking and burning of the granary.

**Table 4:** Control methods used by farmers against storage pests.

Coping strategy	Type of strategy	Communities using it (%)
Chemical pesticides	Chemical control	48.9
Hermetic bag	Hermetic storage	16.2
Ash	Botanical	10.3
Smoking	Traditional	8.7
Proper drying before and during storage	Natural control	6.3
Plastic silos	hermetic storage	4.8
Pepper	Botanical	2.8
Biopesticide (*Lantana camara*)	Botanical	2.4
Neem treatment	Botanical	1.9
Heat treatment	Traditional	0.91
Winnowing	Mechanical control	0.83
Redressing the storage facility	Mechanical control	0.41
Pepper and ash combination	Botanical control	0.33
Use of fern	Botanical	0.25
Bio pesticides	Botanical	0.17
Paraffin	Mechanical control	0.08
Use of tobacco	Mechanical control	0.08
Burning granary	Traditional	0.02
By type	Chemical control	48.9
	Hermetic bag	16.2
	Botanical control	14.5
	Traditional	9.6
	Natural control	6.3
	Plastic silos	4.8
	Mechanical control	1.4

**Fig. 10:**
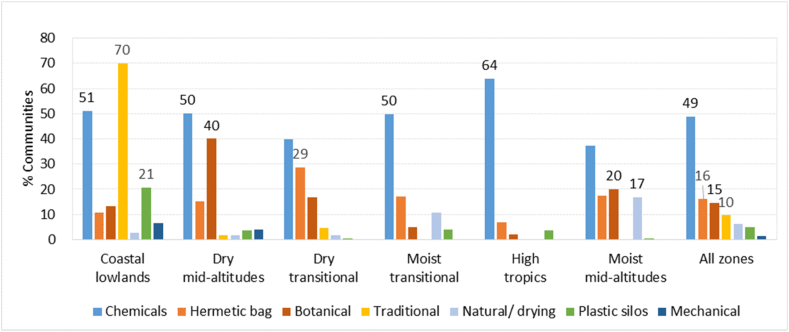
Control methods used by farmers against storage pests by AEZ (in % of communities using).

The popularity of control methods differed by AEZ (Fig. 10). Chemicals were used by more than half of the communities in all zones except the moist mid-altitudes and dry transitional, with the highest levels in the high tropics (64%). Hermetic bags, on the other hand, were more popular in the dry transitional zones (29%) and in the moist mid- and transitional altitudes (17%) but not in the high tropics (7%). Botanical control methods were mainly used in the drylands and were less common in the high tropics. Traditional fire-based methods were mostly used at the coast (by 70% of communities). Natural methods, involving proper drying before storage, were most popular in the humid areas although not used by many communities. Plastic containers were used by 21% of communities at the coast but by less than 5% in all other zones. Other mechanical methods were only used in the coastal lowlands and dry mid-altitudes, and only by a small number of communities.

## 4 Discussion

This study was undertaken to assess farmers' knowledge and observations of storage pests, and to estimate the relative and absolute losses they cause. Our results show that farmers are well aware of storage pests, with almost all participants recognizing maize weevils from the pictures, and most also recognizing the LGB. Farmers’ good knowledge of storage pests has been observed before in Western Kenya (Midega et al., 2016) and Southern Africa (Kamanula et al., 2010). We also find that most farmers were affected by maize weevils, especially in the long rainy season, but less than half were affected by the LGB. In the previous studies, in Wester Kenya LGB affected more farmers than weevils (Midega et al., 2016), while in Malawi both pests affected the same number of farmers and in Zambia weevils affected more farmers (Kamanula et al., 2010).

Relative losses as estimated by participants in the FGDs were high and similar for both species: 21% for weevils and 18% for LGB over the year, or a combined loss of 36%. Relative losses were highest in the humid areas, especially moist mid-altitudes (56%) but lower in the drylands (20–23%). Losses were higher in the long season except in the drylands, where maize production and storage pests were more important in the short season. Total losses in stored maize were estimated at almost 1 million tonnes annually, most of it in the moist mid-altitudes (45%) and the high potential areas (43%). Extrapolating the point data and overlaying them with the maize production map showed the geographic distribution of the losses, with the most important area in western Kenya, around Lake Victoria.

The losses as estimated by FGDs here are not as high as those commonly cited in the early stages of postharvest loss (PHL) studies. Still, the combined losses of both species, 36%, are higher than previous estimates based on physical measurements or synthesis. Early estimates of postharvest food lost in Africa, largely obtained through expert opinion, were typically large round numbers such as 20–40% (Madeley, 1979), 30–50% (Lipton, 1982), or even 40–50% (Zorya et al., 2011), in line with the results of this study. However, physical PHL measurements, including the count and weigh method (Compton et al., 1998) and visual scores of cobs (Compton and Sherington, 1999) led to much lower loss estimates, with an estimate of 4.5% in Kenya (de Lima, 1979). Experimental studies with artificial infestation, finally, found similar results to our study: 19.2% losses from maize weevils and 27.1% from LGB (Edoh Ognakossan et al., 2013).

As these methods are expensive, they are typically only used on small areas, which is problematic because of the high geographic variation. Several reviews tried to synthesize the results of earlier studies. A first review estimated losses at 4%–5% before the arrival of the LGB, but up to 10% afterwards, and even up to 18% under small-scale farming conditions (Tefera, 2012). Similarly, a meta-analysis found PHL in maize in Africa to be between 4% (with interventions) to 21% (without interventions) (Affognon et al., 2015). A synthesis by the African Postharvest Loss Information System (APHLIS found PHL to range between 12% and 20% (Zorya et al., 2011), with a specific estimate for Kenya of 16.7% (https://www.aphlis.net/).

In summary, our estimates of storage losses based on farmer estimates through FGDs are substantially higher than those in the more recent literature, except for the artificial infestation experiments (Edoh Ognakossan et al., 2013). Several possible reasons can be identified. First, in this study farmers were asked to estimate losses from two storage pests separately, and this might have led to double counting; farmers would, for example, estimate relative losses from each species at 50%. Second, farmers’ storage practices change over time, which might increase losses. In Kenya, farmers have moved away from traditional storage in cribs towards storage in bags (Ndegwa et al., 2016). Third, the high geographic variation, and its link with climate as shown in Zambia (De Groote et al., 2023, forthcoming), means that estimates must be taken from a good sample of locations, both sufficiently large and representative of different climates, and this has not been the case in previous studies. Finally, estimates through farmer surveys and FGDs are relatively cheap and can thus solve the problem of representation (hence their recent popularity). Moreover, our experience showed that FGDs can provide farmers with better understanding of pest problems, in particular storage pests. However, the accuracy of loss estimates from farmer surveys and FGDs is not known. These estimates are affected by the way the questions are framed, and asking separately for loss estimates for different insects as was done here might lead to higher estimates than first asking for an overall loss estimate and afterward dividing the loss over different pests, as was done for field pests in Ethiopia (Abro et al., 2021). Methodological studies comparing physical measurements, farmer surveys, and the effect of the way questions are framed, are therefore urgently needed.

## 5 Conclusion

In summary, we find that farmers are well aware and knowledgeable on maize weevils and LGB. Further, we found that most farmers are affected by maize weevils, but less than half were affected by the LGB, Still, estimated relative losses were high for both species: 21% for weevils and 18% for LGB over the year, or a combined loss of 36%, leading to absolute losses of 1 million tonnes of maize annually. We concluded that storage pests remain a major problem, especially in western Kenya, and that the problem might be increasing with changing practices. In particular, few farmers nowadays use traditional methods, but almost half use chemical pesticides. Farmers have started using the more environmentally friendly hermetic bags (16%) and botanicals (15%), which should now be the focus of agricultural extension and agrodealers, especially in the most affected areas identified through this study, in particular Western Kenya.

## Ethical clearance

Ethical clearance for the survey was sought by CIMMYT from CIMMYT's Institutional Research Ethics Committee (IREC), and the research was cleared for implementation on June 11, 2018 (clearance number IREC 2018.004).

## Author statement

HDG and AYB designed the study, developed the tools and supervised data collection; FM analyzed the data; HDG and FM wrote up the results, AYB revised and approved the final manuscript.

## Declaration of competing interest

The authors declare that they have no known competing financial interests or personal relationships that could have appeared to influence the work reported in this paper.

## Data Availability

The data used in this paper are uploaded on the CIMMYT repository and are freely available (De Groote et al., 2022).

## Appendix A Supplementary data

Mutimedia component 1 The questionnaire of the FGD is found in Supplementary Material 1. The data used in this paper are uploaded on the CIMMYT repository and are freely available (De Groote et al., 2022). Mutimedia component 1
