# Supplementary material for: On-farm storage loss estimates of maize in Kenya using community survey methods
Source: J Stored Prod Res. 2023 May;102:102107. doi: 10.1016/j.jspr.2023.102107 (PMC10285508; doi:10.1016/j.jspr.2023.102107)
Supplement: Mutimedia component 1 — The questionnaire of the FGD is found in Supplementary Material 1. The data used in this paper are uploaded on the CIMMYT repository and are freely available (De Groote et al., 2022). [file mmc1.pdf]

KENYA COMMUNITY SURVEY (2018)

Objectives

The purpose of this community survey is to guide the Stress Tolerant Maize for Africa (STMA) project in understanding biotic and abiotic stresses in small holder maize producing households in the different agro ecological zones, and understand their maize seed purchase behavior in function of those stresses.

COMMUNITY IDENTIFICATION

	Zone	Sub-location
**CID**		

3-digit code: zone (1digit), sub-location (2 digits)

			Name	Code
Date visit (DD/MM/YYYY)				
Time start (HH:MM)		Facilitators		
Date entered (DD/MM/YYYY)		Entered by:		

Variable	Response	Code (provided)
AEZ		
County		
District/Sub-county		
Division		
Location		
Sub-location		
Village name		
GPS Coordinates		

INFORMED CONSENT INFORMATION SHEET

International Maize and Wheat Improvement Centre (CIMMYT) and Agri-Food Economics Africa

Kenya Community Survey

To the Facilitator: *Convey the complete information below to the members of the focus group. Then ask the participants if they have any questions, and answer these questions accordingly. If the participants agree to participate in the focus group discussion, please document their verbal informed consent.*

You are being asked to participate in a research study. A member of the research team will describe the study to you and answer all your questions. Please listen to the information and ask questions about anything you do not understand before deciding whether or not to participate.

Why is this study being done?
This study will provide information to the Stress Tolerant Maize for Africa (STMA) project in understanding biotic and abiotic stresses in small holder maize producing households. This understanding is important in guiding research on the development of new technologies that can cope with such stresses. For agricultural research to be successful, it needs to respond to farmers' demands and fit farmers' priorities. It is therefore important that research focusses on what farmers see as their priority constraints. The results of this survey will therefore contribute to our understanding of these constraints. The decision on your involvement will be made by you and only you.

What happens if you participate in this study?
If you participate in this study, you will be a part of a group of community members who will be asked questions on your community's pests and other farmers' constraints.

Risks or discomforts
You or the group will not be exposed to any risks by participating in this study.

Benefits
The results of this study will be used to guide research priorities for the STMA Project and can provide useful information for the implementation of future interventions to address farmer constraints and improve agriculture in Kenya.

Confidentiality
All information about your community will be kept strictly confidential. You or the group will not be personally identified in any study report or publications. Data may be shared with other CIMMYT researchers. However, we are committed to ensuring absolute confidentiality

Participation
Participation in this study is voluntary. You have the right to refuse to participate in this study. If you choose to participate, you have the right to stop at any time and to not answer certain questions. If you refuse or stop your participation at any time, there will be no consequences.

Questions
You may ask any questions you have about the study. If you have questions later, they can be directed to Dr. Hugo De Groote, Principal Scientist, International Maize and Wheat Improvement Center (CIMMYT), P.O. Box 1041 Village Market-00621, Nairobi Kenya. Telephone +254 20 722 4604

Informed consent given? ___________ **(Y/N)**

Summary of Focus Group Participants

Gender	No. aged 18-35 years	No. aged 36-59 years	No. aged 60 years and older	Total
Women				
Men				

SECTION 1: WOMEN AND MEN TOGETHER

1. MODULE 1: GENERAL INTRODUCTORY QUESTIONS

1.1. How many households (HH) are in this community? ____________________

1.2. Which share/percentage of the households rely on the agricultural sector and its sub-sectors as their **MAIN** livelihood activity? _________________________

1.3. What are the main crops grown in this community? *(list the 5 most commonly grown, starting with mostly commonly grown)(Note to enumerator: list crops in a notebook and probe on the rank)*

No	Crop	Is this crop mainly intercropped (1) or mono-cropped (0)?	Main intercrop crop (if mainly intercropped)	How many times per year is this crop normally grown? *(Fill 99 for perennial crops)*
**1.**				
**2.**				
**3.**				
**4.**				
**5.**				

SECTION 2: GENDER DISAGGREGATED

Gender: _____________ (Male/Female)

2. COMMONLY GROWN MAIZE VARIETIES AND YIELD

2.1. Which maize varieties do you know that are commonly grown in this community? *(Name 10).*

**No.**	**Varieties Codes** *(For local variety, fill code 777 and specify; for other variety that is not local, fill code 888 and specify)*
**1.**	
**2.**	
**3.**	
**4.**	
**5**	
**6.**	
**7.**	
**8.**	
**9.**	
**10**	

(Probe for STMA varieties – see variety list attached)

2.2. Which seasons is maize normally grown in this community?

- March planting (Y/N)
- October planting (Y/N)

2.3. What is the average maize plot sizes and production in this community? *(In acres)*

**Season**	**Average maize plot size (Acres)**	**Average production (90kg bags)**	**Calculate yield (90kg bags/acre)**
March planting			
October planting			

3. SCORING OF BIOTIC AND ABIOTIC STRESSES

3.1. What are the main constraints in relation to maize production in this community? *(Show pictures for each constraint mentioned before asking importance and trend)*

**Constraint *(specific)***	Importance *(on a scale of A - E, with A being most important)*	Trend of the constraint in the last five years *(-1 = decreasing; 0 = same; 1 = increasing)*
1.		
2.		
3.		
4.		
5.		

(If not mentioned, probe for importance of wild/domestic animals' damage; drought)

3.2. What are the main constraints in relation to maize storage in this community? *(Show pictures for each constraint mentioned before asking importance and trend)*

**Constraint *(specific)***	Importance *(on a scale of A - E, with A being most important)*	Trend of the constraint in the last five years *(-1 = decreasing; 0 = same; 1 = increasing)*
1.		
2.		
3.		
4.		
5.		

(If not mentioned, probe for importance of rodents)

4. FALL ARMYWORM (FAW) PEST IN KENYA

(only use this as a guide, do not read):

The Fall Army Worm *(Spodoptera frugiperda*) is an insect pest of the moth family of which the larvae attack many crops including maize, rice, sorghum, wheat, and vegetable crops. It reproduces at a rate of several generations per year, and the moth can fly up to 100 km per night. It originated from the tropical Americas and was first reported in Africa in 2016.

4.1. How many can correctly identify the FAW *(use the local language name for FAW)*?

Show pictures (Picture 1 or 2 depending on which stemborer is prominent in that area) of larvae of FAW, African Army Worm and Stemborer and record the number that says that is FAW

**Pictures of Insect Stages**	**Number saying this is FAW**
Picture A	
Picture B	
Picture C	

(Show correct picture of FAW plus (Picture 3) its damage and ask the following questions)

4.2. Has FAW ever been observed in this community? 1 = Yes (go to 4.3), 0 = No (go to next module): ___________________

4.3. If **Yes**, which year did it first occur in this community? ______________________ *(if they don't know because it is too long ago, put 999)*

4.4. Has FAW been observed in this community in the following seasons?

Season	Observed in this season (Y/N)	% of farmers affected in this community	% yield reduction due to FAW *(estimate for current season)*
March 2018 planting *(current)*			
Oct 2017 planting			

March 2017 planting			

4.5. Which crops were most affected in last 3 seasons? *(mention 5 starting with the most severely affected)*

**Season**	**Crop**	**At what stage did you start observing the FAW** *(1 = emergence/seedling/ early stage; 2 = vegetative/ (before flowering); 3 = flowering/tasseling; 4 = post flowering/late stage)5=cob/grain*	**In comparison to an average year, what was the condition of your plants just before the FAW attack?** (1=Good, 2=Average; 3=Poor)
March 2018 planting *(current)*	1.		
	2.		
	3.		
	4.		
	5.		
October 2017 planting	1.		
	2.		
	3.		
	4.		
	5.		
March 2017 planting	1.		
	2.		
	3.		
	4.		
	5.		

4.6. How is the attack from FAW this year compared to last year? (-1 = less, 0 = same, 1 = more)

4.7. What are the common methods used here currently to control FAW and how effective are they in reducing loss? *(Name 5 by level of popularity – see options below. Probe for local/traditional methods)*

**No.**	**Method**	**Proportion of farmers using (%)**	**Effectiveness** *(from A to E, with A being most effective)*
1.			
2.			
3.			

4.			
5.			

1. Pesticides application
2. Remove crop residues that are damaged by pests
3. Applying powder washing soap
4. Apply ash on young larvae
5. Early planting
6. Late planting
7. Replanting
8. Plant resistant varieties/tolerant varieties
9. Crop rotation with non-host plants e.g. legumes
10. Frequent weeding (to remove alternative host plants)
11. Improve soil fertility – manure or chemical fertilizer
12. Application of biopesticides e.g. neem-based products
13. Intercrop maize with legumes
14. Intercropping maize with non-legume crops
15. Trap cropping – use a trap plant e.g. Napier grass as a border crop
16. Push-pull – use a trap plant as a border crop and intercrop with a repellent plant
17. Uprooting and burning of infected plants
18. Handpicking egg masses and caterpillars
19. Biological control options (encouraging natural enemies to enter the field by planting their host plants in the field boundaries)
20. Biorational control (virus, fungi, bacteria, nematodes)
21. Pheromones usage
22. Combinations (specify)
23. Other1 (specify)
24. Other2 (specify)
25. Other3 (specify)

4.8. If planting resistant maize varieties is mentioned as a control method, which are most common resistant varieties planted? *(Name 3)*

4.9. If application of pesticides is mentioned as a control method, which are most common pesticides applied and what are their effectiveness? *(Name 3)*

Pesticide	**Effectiveness** *(from A to E, with A being most effective)*
1.	
2.	
3.	

4.10. What support have people in this community received on FAW and who provided the support?

**Category**	**Specific support**	**Main provider**
Information/ training/extension	1.	
	2.	
	3.	
	4.	
	5.	
Pesticides	1.	
	2.	
	3.	
	4.	
	5.	

4.11. What other support are needed in this community so as to cope better with FAW?

**Category**	**Specific support**	**Score in terms of importance** *(from A to E, with A being most important)*
Information/ training/extension	1.	
	2.	
	3.	
	4.	
	5.	
Pesticides	1.	
	2.	
	3.	
	4.	
	5.	
Others	1.	

	2.	
	3.	
	4.	
	5.	

5. **ACCESS TO SEED**

5.1. Do farmers in this community face constraints in accessing maize seed? *(Score the 5 main constraints)*

**Constraint**	**Score** *(from A to E, with A being most important)*

Time at this point: _________

SECTION 3: WOMEN AND MEN TOGETHER

6. **AREA ALLOCATED TO DIFFERENT MAIZE**

6.1. What is the average number of maize varieties planted per farmer in this community?

Aggregate the maize varieties identified separately by women and men (in section 2) into one list and ask:

6.2. What proportion of farmers are growing different maize varieties in this community?

**Variety**	**Farmers growing variety (%)**
**1.**	

**2.**	
**3.**	
**4.**	
**5.**	
**6.**	
**7.**	
**8.**	
**9.**	
**10.**	
**11.**	
**12.**	
**13.**	
**14.**	
**15.**	

6.3. For the 3 varieties grown by the greatest % of farmers, which attributes make them the most popular? *(Rank the main 3 reasons starting with the most important)*

**Maize variety**	**Reason 1**	**Reason 2**	**Reason 3**
**1.**			
**2.**			
**3.**			

7. MAIZE LETHAL NECROSIS (MLN) DISEASE IN KENYA

(Only use this as a guide, do not read):

According to Dr Dan Makumbi (2013),
"MLN is a serious disease of maize which appeared in eastern Africa farmers' fields in 2011. It is caused by a combination of two viruses that are difficult to differentiate individually based on visual symptoms. The insects that transmit the disease-causing viruses may be carried by wind over long distances

(Show pictures (Picture 4) and ask the following questions)

7.1. How many of you know the Maize Lethal Necrosis? ___________

7.2. Has the disease been observed in this community? 1 = Yes (go to 7.3), 0 = No (go to next module) ___________________

7.3. If Yes, which year did it first occur in this community? _______________________ *(if they don't know because it is too long ago, put 999)*

7.4. Has MLN been observed in this community in the following seasons?

Season	Observed in this season (Y/N)	% of farmers affected in this community	% yield reduction due to MLN *(estimate for current season)*
March 2018 planting *(current)*			
Oct 2017 planting			
March 2017 planting			

7.5. Over the last 5 years, has MLN attack been decreasing or increasing? (-1 = decreasing, 0 = same, 1 = increasing) __________________________________

7.6. If it has been decreasing, what was the peak attack year? __________________________

7.7. What are the common methods used here to control the disease and, how effective are they in reducing loss? *(Name 3 by level of popularity)*

	**Method**	**Proportion of farmers using (%)**	**Effectiveness (from A to E, with A being most effective)**
1.			
2.			
3.			

7.8. Are there any resistant varieties to MLN? If so, which ones? *(Name 3)*

	**Resistant variety to MLN**	**Main Reason why this variety is not commonly grown in this community?** (*If commonly grown, write "grown"). If reason not known, write "don't know"*
1.		
2.		
3.		

If not mentioned, probe for H12ML, WE5135MLN, and DK777

8. MAIZE STEMBORER IN KENYA

(only use this as a guide, do not read):

"Stemborer is…describe definition for training purposes.."

(show pictures (Picture 5 or Picture 6) and ask the following questions)

8.1. How many of you know the maize Stemborer? ___________

8.2. Has the pest been observed in this community? 1 = Yes (go to 8.3), 0 = No (go to next module)______________

8.3. If Yes, which year did it first occur in this community? ________________ *(if they don't know because it is too long ago, put 999)*

8.4. Has stemborer been observed in this community in the following seasons?

Season	Observed in this season (Y/N)	% of farmers affected in this community	% yield reduction due to stemborer *(estimate for current season)*
March 2018 planting *(current)*			
Oct 2017 planting			
March 2017 planting			

8.5. Over the last 5 years, has attack by Stemborer been decreasing or increasing? (-1 = decreasing, 0 = same, 1 = increasing)

8.6. If it has been decreasing, what was the peak attack year in the last 5 years? ___________

8.7. What are the common methods used here to control Stemborer and how effective are they in reducing loss? *(Name 3 by level of popularity)*

No.	Method	Proportion of farmers using (%)	Effectiveness (from A to E, with A being most effective)
1.			
2.			
3.			
If not mentioned, probe for the following as control methods			
1.	Push pull		
2.	Insect-resistant varieties (Probe for resistant varieties)		
3.	Pesticides		
4.	Bio-pesticides (e.g. Neem, *bt*, pesticides)		

9. STRIGA WEED IN KENYA

(only use this as a guide, do not read):

Striga is a parasitic weed that attacks cereal crops and legumes, retarding plant growth, resulting in stunted and withered plants. S*triga* attaches itself to the roots of host plants and siphons the nutrients and water intended for plant growth. This stunts and discolours the plant, finally causing it to wither resulting in grain yield losses. In cereals, there are two species of economic importance: one has purple flowers (*S.* hermonthica), the most destructive one, and one has red flowers (*S. asiatica).*

(show pictures (Picture 7) and ask the following questions)

9.1. How many of you know Striga weed? __________

9.2. Has this weed been observed in this community? 1 = Yes (go to 9.3), 0 = No (go to next module)_____________

9.3. If Yes, which year did it first occur in this community? _______________ *(if they don't know because it is too long ago, put 999)*

9.4. Has Striga been observed in this community in the following seasons?

Season	Observed in this season (Y/N)	% of farmers affected in this community	% yield reduction due to Striga *(estimate for current season)*
March 2018 planting *(current)*			
Oct 2017 planting			
March 2017 planting			

9.5. Over the last 5 years, has the attack by Striga weed been decreasing or increasing? (-1 = decreasing, 0 = same, 1 = increasing)

9.6. If it has been decreasing, what was the peak attack year? ___________

9.7. What are the common methods used here to control the weed and how effective are they in reducing loss? *(Name 3 by level of popularity)*

No.	**Method** *(see options below)*	**Proportion of farmers using (%)**	**Effectiveness (on a scale of A-E, with A being most effective)**
1.			
2.			
3.			

Probe for resistant varieties

1. Push pull
2. IR Maize
3. Herbicides
4. Mechanical weeding
5. Others, specify

10. MAIZE WEEVIL IN KENYA

(only use this as a guide, do not read):

"Maize Weevil…describe definition for training purposes.."

(show pictures (Picture 8) and ask the following questions)

10.1. How many of you know the maize weevil? ____________

10.2. Has the pest been observed in this community? 1 = Yes (go to 10.3), 0 = No (go to next module)____________

10.3. If Yes, which year did it first occur in this community? __________________ *(if they don't know because it is too long ago, put 999)*

10.4. Has the Maize Weevil been observed in this community in the following seasons?

Season	Observed in this season (Y/N)	% of farmers affected in this community	% storage loss due to Maize weevil
March 2018 planting *(current)*			
Oct 2017 planting			
March 2017 planting			

10.5. Over the last 5 years, has attack by maize weevil been decreasing or increasing? (-1 = decreasing, 0 = same, 1 = increasing) ____________________________

10.6. If it has been decreasing, what was the peak attack year? __________

11. LARGER GRAIN BORER (LGB) IN KENYA

(only use this as a guide, do not read):

"LGB…describe definition for training purposes.."

(show pictures (Picture 9) and ask the following questions)

11.1. How many of you know the LGB? ____________

11.2. Has the pest been observed in this community? 1 = Yes (go to 11.3), 0 = No (go to next module)_____________

11.3. If Yes, which year did it first occur in this community? _______________ *(if they don't know because it is too long ago, put 999)*

11.4. Has the LGB been observed in this community in the following seasons?

Season	Observed in this season (Y/N)	% of farmers affected in this community	% storage loss due to LGB
March 2018 planting *(current)*			
Oct 2017 planting			
March 2017 planting			

11.5. Over the last 5 years, has attack by LGB been decreasing or increasing? (-1 = decreasing, 0 = same, 1 = increasing)_______________________________

11.6. If it has been decreasing, what was the peak attack year? ___________

Show picture of storage insect pests and ask the following:

11.7. What are the common methods used here to control storage insect pests and how effective are they in reducing loss? *(Name 3 by level of popularity)*

**No.**	**Method**	**Proportion of farmers using (%)**	**Effectiveness (on a scale of A - E, with A being most effective)**
1.			
2.			
3.			
If not mentioned, probe for the following as control methods			
1.	Hermetic bags		
2.	Metal silos		
3.	Plastic silos		
4.	Insecticides		
5.	Biological agents (specify)		

11.8. If pesticides are mentioned as a control method, which are the most common pesticides applied and what are their effectiveness? *(Name 3)*

Pesticide	Effectiveness *(from A to E, with A being most effective)*
1.	
2.	
3.	

11.9. Which maize varieties are known to be resistant to storage pests in this community? *(Name 3)*

12. DROUGHT

12.1. How important is drought as a constraint to maize production in this community *(on a scale of A - E, with A being most important)*

12.2. In the last 5 years, in which years did this community experience drought?

**Year**	**Drought experienced?** *(Yes/No)*
2017	
2016	
2015	
2014	
2013	

12.3. What are the common drought coping strategies in this community? *(Mention 3; probe for water harvesting technologies and resistant varieties)*

13. SOIL FERTILITY

13.1. How important is poor soil fertility as a constraint to maize production in this community *(on a scale of A - E, with A being most important)*

13.2. By how much has poor soil fertility reduced production in this community?

13.3. What are the common practices to cope with poor soil fertility in this community? *(Probe for nitrogen efficient varieties)*

13.4. What is the percentage of farmers using fertilizer in maize production this community?

13.5. Did farmers in this community receive subsidized fertilizer? *(Note: this refers to fertilizer for general crop use: not those specific for tea,.)* _________(Y/N)

13.6. If yes, what percentage of farmers in this community received subsidized fertilizer?

13.7. What is the main source of the subsidized fertilizer?

13.8. What difference did the subsidized make? *(on a scale of A - E, with A meaning it made a lot of difference)*

Community contacts *(get at least 2 contacts)*:

a) Name: ____________________________________ Tel: _________________________

b) Name _____________________________________ Tel: _________________________

TIME FINISHED (HH:MM) ________________

Appendix: Picture of biotic stresses

Picture 1: Gauging if participants can correctly identify FAW (with *Busseola fusca)*

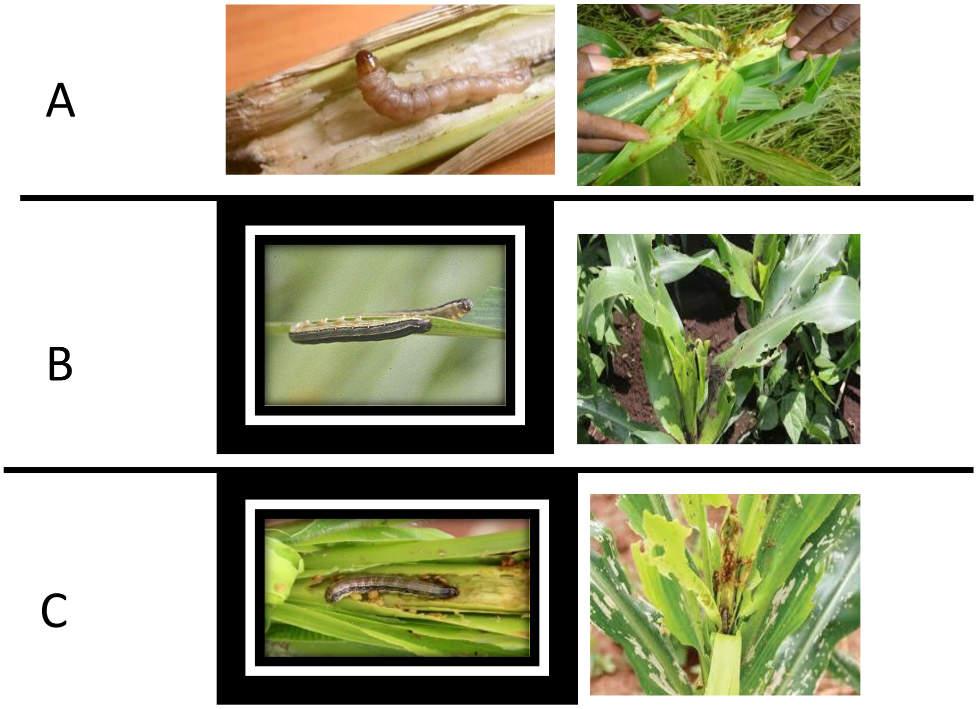

Picture 2: Gauging if participants can correctly identify FAW (with *Chilo partellus)*

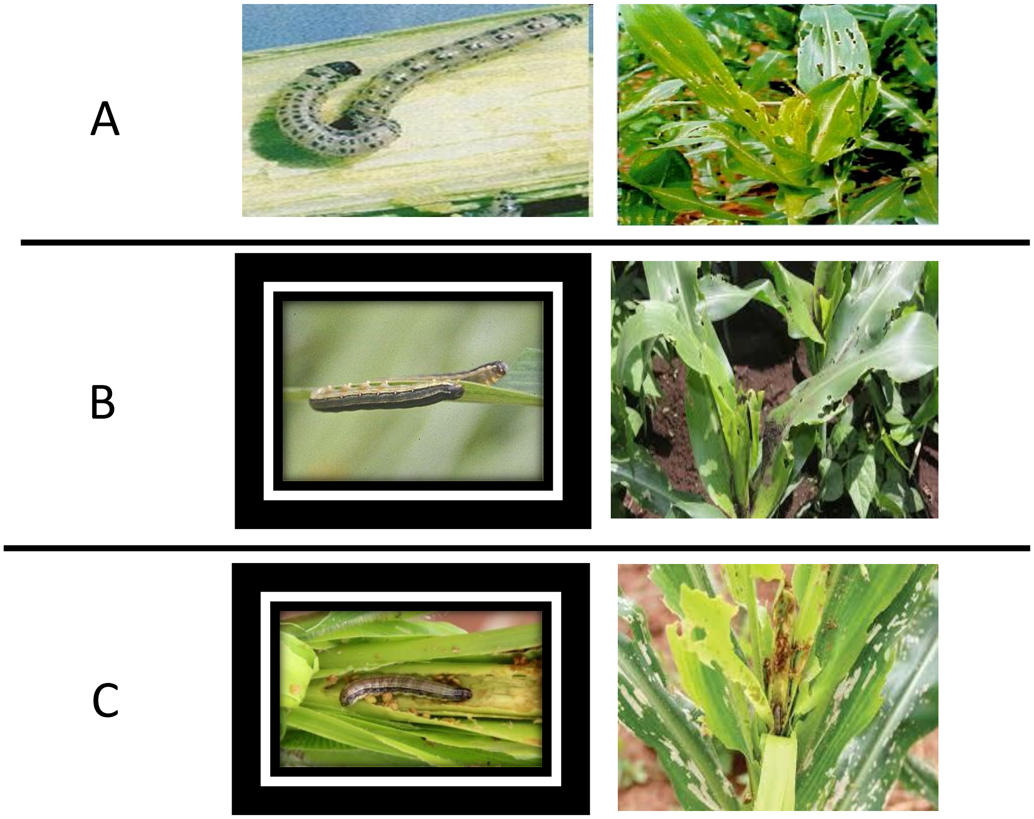

Picture 3: Fall Army Worm

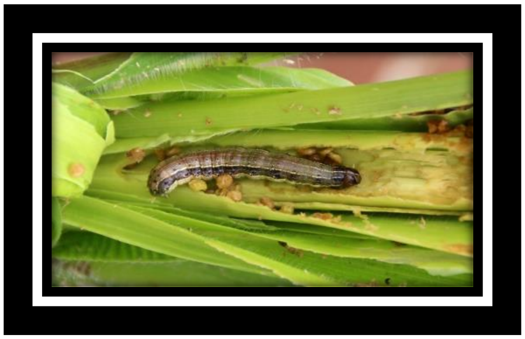 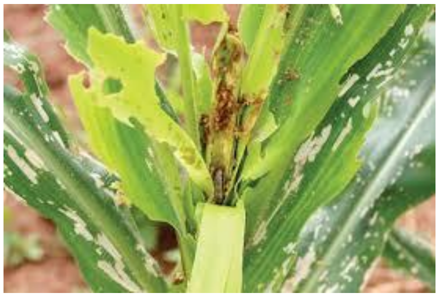

Picture 4: MLN

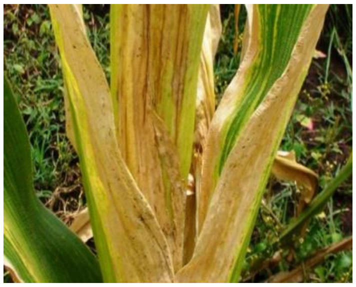 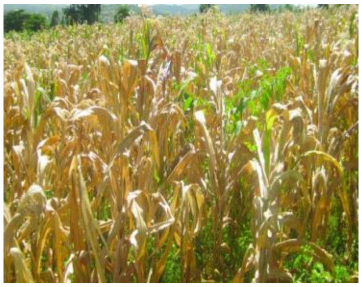

Picture 5: Maize Stemborer (BF)

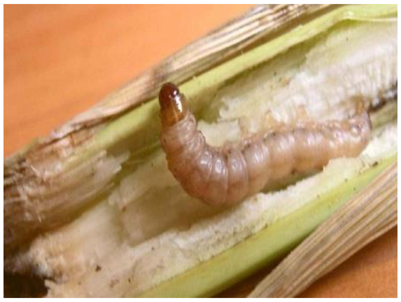 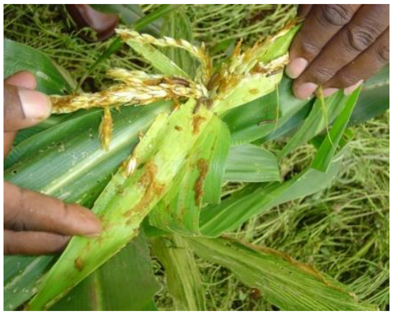

Picture 6: Maize Stemborer (CP)

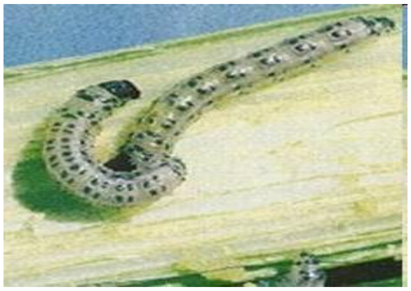 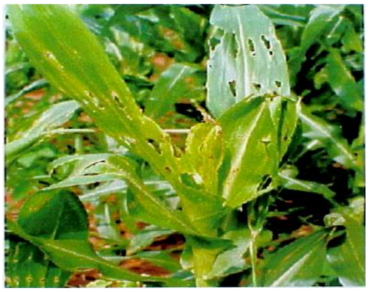

Picture 7: Striga Weed

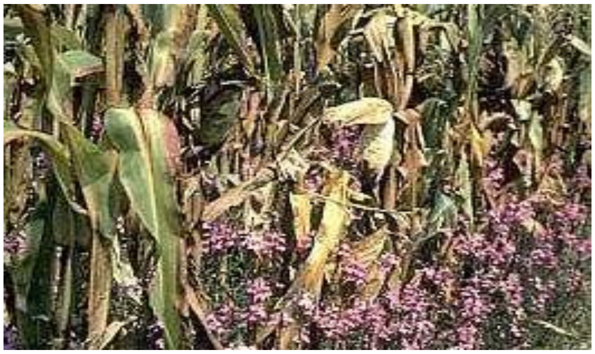

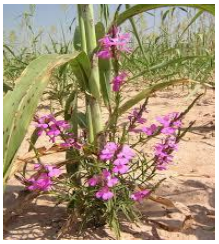

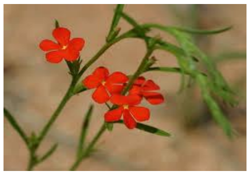

Picture 8: Maize Weevil

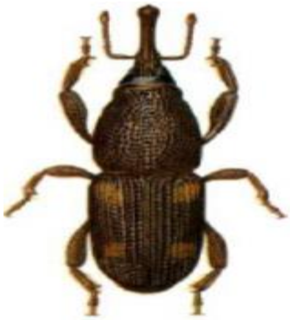

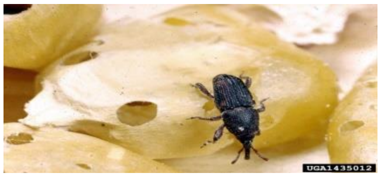

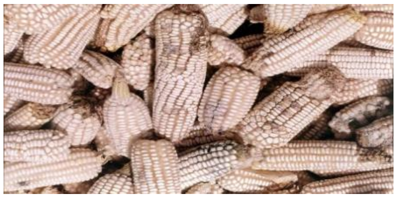

Picture 9: Larger Grain Borer

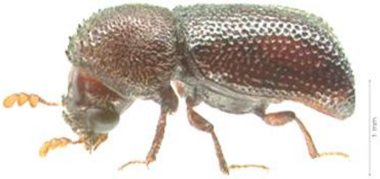

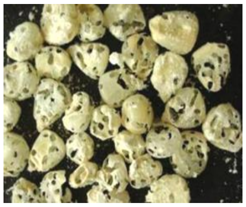

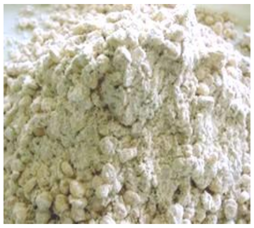

For further information, contact Simon Kimenju at skimenju@agrifoodeconomics.com or Hugo De Groote at h.degroote@cgiar.org
